# *In silico* analysis and *in vivo* tests of the tuna dark muscle hydrolysate anti-oxidation effect†

**DOI:** 10.1039/c8ra00889b

**Published:** 2018-04-17

**Authors:** Jiaojiao Han, Shasha Tang, Yanyan Li, Wei Bao, Haitao Wan, Chenyang Lu, Jun Zhou, Ye Li, Lingzhi Cheong, Xiurong Su

**Affiliations:** School of Marine Science, Ningbo University 818 Fenghua Road Ningbo China suxiurong_public@163.com zhoujun1@nbu.edu.cn +86 574 87608368 +86 574 87608368; College of Agriculture and Life Sciences, Cornell University Ithaca USA

## Abstract

Hydrolysate is a mixture of various peptides with specific functions. However, functional identification of hydrolysate with high throughput is still a difficult task. Furthermore, using *in vivo* tests *via* animal or cell experiments is time and labor-intensive. In this study, the peptides component of hydrolysate derived from the tuna dark muscle was measured *via* MALDI-TOF/TOF-MS, and the functions of the KEFT (Lys-Glu-Phe-Thr), EEASA (Glu-Glu-Ala-Ser-Ala) and RYDD (Arg-Tyr-Asp-Asp) peptides, which were found with the highest proportion, were predicted *via* Discovery Studio 2016 software. All three peptides were predicted to bind to the Keap1 protein with the highest fit-value and to affect the activity of Keap1, which is involved in anti-oxidation pathways. Subsequently, mice experiments showed that administration of tuna dark muscle hydrolysate increased the levels of superoxide dismutase and glutathione peroxidase in the serum and liver (*P* < 0.05) and decreased the malondialdehyde level (*P* < 0.05) as well as transcription of Keap1 (*P* > 0.05), which are consistent with the *in silico* analysis results using Discovery Studio 2016 software. The combination of *in silico* analysis and *in vivo* tests provided an alternative strategy for identifying hydrolysate function and provided insight into high-value utilization of protein hydrolysate.

## Abstract 2

*In silico* prediction and *in vivo* confirmation of anti-oxidation effect.

## Graphical abstract

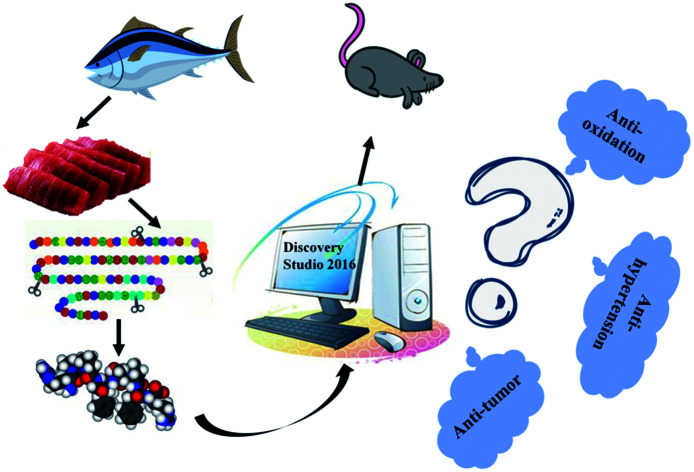

## Introduction

Redox reactions, the most important metabolic pathway of life, are necessary for biological energy.^1^ However, oxidative stress is also considered to be an important risk for diseases, such as diabetes mellitus, cancer, cardiovascular disease and Alzheimer's disease.^2^ Antioxidants are substances that prevent the adverse effects of oxidative stress. Currently, synthetic antioxidants occupy an important position due to their strong antioxidant activity. However, synthetic antioxidants such as butylated hydroxytoluene and butyl-hydroxyanisole, will lead to liver enlargement, microsomal enzyme activity enhancement, and carcinogenicity. As a result, the use of synthetic antioxidants has been strictly regulated. Therefore, it is necessary to develop other safe and effective antioxidant candidates.

Several studies have demonstrated that marine protein hydrolysates are abundant natural sources of antioxidants. An antioxidative peptide extracted from the enzymatic hydrolysate of yellowfin sole possesses antioxidative activity that is 3-fold greater than that of α-tocopherol.^3^ Peptides extracted from giant kingfish *Caranx ignobilis* exhibited strong antioxidant activities and were evaluated according to their 2,2-diphenyl-1-picrylhydrazyl radical scavenging activity, reducing power and metal chelating activity. In addition, two types of peptides extracted from the *Rastrineobola argentea via* different enzyme treatments were shown to have strong antioxidant activities of 31.5% and 49.5% by using oleic acid (lipid) peroxidation inhibition to measure their antioxidant potential. Meanwhile, the corresponding data for the commercial antioxidant butyl-hydroxytoluene is 29%.^4^

Tuna belongs to the Osteichthyes, Perciformes, and Scombridae classes and is a highly migratory fish that lives in the middle of bodies of water. Tuna is well appreciated worldwide because of its high nutritional value and potential health benefits.^5^ However, large quantities of fish protein (especially the dark muscle, 50–70% of the raw material) are regarded as by-products and are discarded without any attempt at recovery or valorization. Studies have shown that tuna peptides have various functions. Peptides isolated from the tuna backbone protein have strong antioxidant activity,^6^ and peptides obtained from the tuna cooking juice have angiotensin I-converting enzyme (ACE) inhibition activity that can be used to lower blood pressure.^7^ Dark muscle contains abundant protein and is a promising and potential alternative protein source for the preparation of peptides. However, the enzymatic hydrolysate is a mixture of various peptides, and functional identification of the hydrolysate with high throughput still a hard task, whereas one-by-one *in vivo* tests *via* animal or cell experiments are time and labor-intensive.

Discovery Studio 2016 (DS 2016) is a new generation of molecular modeling software that is mainly used in protein structure and drug discovery.^8^ Reverse docking is a method that does the opposite of virtual screening by using docking.^9^ The concept of one-ligand with many targets is an important tool for the drug development.^10^ Molecular docking is based on geometric matching and energy matching to find the best match mode between receptors and ligands.^11^ The DS3.1 LigandFit and CDOCKER docking programs were used to obtain a preliminarily estimate of the binding sites of α-cyperone to human serum albumin.^8^ To explore the structural-functional mechanism between purified peptides (Tyr-Arg and Ile-Arg) and ACE, DS3.0 was used to simulate computational molecular docking.^12^ Recently, traditional cholesterol-lowering statins were predicted to have anti-tumor activity *via* Discovery Studio software, and this prediction was subsequently confirmed by cell experiments. The main ingredients, ginseng and ginsenosides, were evaluated for anti-cancer properties *via* reverse docking.^13^ In addition, ganoderma secondary metabolites were found to have antiviral activity by *in silico* profiling,^14^ and the simulation results were verified by *in vitro* experiments. Previous studies indicated the peptides can be used as ligands in the Discovery Studio,^15^ and the target proteins binding to the peptide ligands might provide information about the function of the peptides, as well as the hydrolysate (peptides mixture).

In consideration that the traditional experiment-and-screen methods suffer from being time and labor-intensive, a strategy combining *in silico* analysis and *in vivo* tests to screen the functions of hydrolysate with high throughput is provided in this study. After the determination of optimal enzymatic hydrolysis conditions with single factor and response surface experiments, the majority peptides in the hydrolysate are measured, the functions are subsequently predicted *via* software Discovery Studio 2016, and further verified *via* animal experiments.

## Materials and methods

### Optimization of enzymatic hydrolysis conditions

As previously described, the optimal enzymatic hydrolysis conditions were determined *via* single factor and response surface experiments, including the combination of the enzyme type, hydrolysis temperature, hydrolysis time, and enzyme and solid–liquid ratio content.^16^ The detailed process for optimizing the enzymatic hydrolysis conditions is described in the ESI.†

### Peptide identification

Hydrolysation of tuna dark muscle (Ningbo Today Food Co., Ltd, Ningbo, China) was performed under optimal conditions as described previously. After 10 min inactivation with boiling water, it was cooled to room temperature and the solution was centrifuged at 4000 rpm at 4 °C for 10 min to obtain a precipitate (Thermo Scientific, MA, USA). Then, polypeptides were separated by ultrafiltration. The low molecular weight polypeptides were obtained using ultrafiltration membrane Millipore 8050 (Millipore, MA, USA) and stored in a vacuum freeze-drying machine (Free Zone 2.5 L, LABCONCO Co., Ltd, Kansas City, USA) for 24 h.

As previously described, the hydrolysate was resuspended in 60 μL of methanol/water (50/50, v/v) with 0.2% formic acid and 2 mM sodium acetate prior to matrix-assisted laser dissociation time-of-flight mass spectrometer analysis^17^ (MALDI-TOF/TOF AB SCIEX, Applied Biosystems, CA, USA). The sample (0.5 mL) was directly spotted on a MALDI plate and mixed with an equal volume of 2.5 dihydroxybenzoic acid-matrix prepared by suspending 10 mg of 2.5 dihydroxybenzoic acid in 1 mL of a water/methanol (50/50, v/v) solution containing 1 mmol l^−1^ sodium acetate. The MALDI plate was dried under vacuum to ensure uniform crystallization. Mass spectra were acquired using a 5800 MALDI TOF/TOF Analyzer (AB SCIEX, Applied Biosystems, CA, USA).^18^ The samples were collected and analyzed by MASCOT^19^ and NCBI to obtain the structure of the polypeptide. The precise molecular weight was determined by primary mass spectrometry, and the amino acid sequence was determined by secondary mass spectrometry.^20^

### Reverse molecular docking based on ligands

The ligand Profiler and CDOCKER docking programs implemented in Discovery Studio 2016 software (Beijing Chong Teng Technology Co., Ltd, Beijing, China) were used in this study. First, the structures of the peptides were constructed using Discovery Studio 2016 software. The structure was energy minimized using the steepest descent and conjugate gradient techniques. Then, the “Prepare Ligands” program in Discovery Studio was used to generate multiple conformations. This process was performed using the DS Ligand Profiler tool. In this work, we used the Pharma DB as our target database. During the procedure, the parameters were set to the shape of PharmaDB pharmacophores, to the most selective of the input PharmaDB pharmacophores, and to be true for save aligned ligands. The other parameters were set to the default values.

Pharmacophore crystal structures with fit-value > 3 were obtained from the protein data bank (PDB) for docking simulations. The procedure was performed using the CDOCKER tool. During the procedure, the proteins were downloaded from the PDB as receptors, and the peptides were set as ligands. The ligand–receptor complex energy scores determined whether the ligands and receptor proteins bonded.

### Animal experiment design

All of the experimental procedures and animal care were performed in accordance with the Guide for the Care and Use of Laboratory Animals prepared by the Ningbo University Laboratory Animal Center (affiliated with the Zhejiang Laboratory Animal Common Service Platform), and all of the animal protocols were approved by the Ningbo University Laboratory Animal Center under permit number no. SYXK (ZHE 2008-0110).

After 2 weeks of acclimatization, twenty-four 10 week-old male ICR mice (23.5 ± 2.3 g, Laboratory Animal Center of Zhejiang province, Zhejiang, China, SCXK (Zhejiang) 2014-0001 no. 1605200003) were randomly divided into a control group and an experimental group. The mice in the experimental group were gavaged with 300 mg kg^−1^ d^−1^ tuna dark muscle hydrolysate,^21^ and the mice in the control group were given the same amount of normal saline. The twelve mice in each group were housed in three cages (four per cage). During the experiment, mice had free access to water and feed, and room temperature was maintained at 23–25 °C; night and day intervals were set to 12 h.

Body weight was measured every three days. After 40 days of feeding, all animals were anaesthetized by enflurane. Then, blood samples were taken from the orbital plexus. We separated serum *via* centrifugation for 15 min at 4 °C at 3000 rpm and stored it at −80 °C. Next, all animals were sacrificed by cervical dislocation. For further analysis, tissues, including the liver and brain, were weighed, and immediately frozen in −80 °C.

### Physiological and biochemical indexes

Biochemical measurements of TC (total cholesterol), TG (triglyceride), HDL (high-density lipoprotein), LDL (low density lipoprotein), SOD (superoxide dismutase), GSH-Px (glutathione peroxidase) and MDA (malondialdehyde) were measured *via* kits (Nanjing Jiancheng Bioengineering Institute, Nanjing, China) according to the manufacturer's procedure.

### Quantitative real-time PCR

As previously described,^22^ total RNA from frozen liver and brain was extracted using TransZol Up Plus RNA Kit (TRAN, Beijing, China). Residual genomic DNA was removed using RNase-free DNase I (MBI Fermentas, Vilnius, Republic of Lithuania) according to the manufacturer's protocol. The RNA concentration was determined using a NanoDrop 2000 UV-Vis spectrophotometer (Thermo Scientific, MA, USA). Subsequently, reverse transcription was achieved using total RNA as the starting material and the TransScript All-in-One First-Stand cDNA kit (TRAN, Beijing, China). The transcriptional levels of genes were determined by quantitative real-time RT-PCR (qRT-PCR) performed on a Rotor-Gene 6000 realtime PCR machine (Corbett, Australia) with SYBR® Premix Ex TaqTM II according to the manufacturer's protocols.^22^ The samples were run at 95 °C for 5 min, followed by 40 cycles of 15 s denaturation at 95 °C, 30 s annealing at 55 °C and elongation for 30 s at 72 °C, followed by melting curve analysis.

Target genes were selected based on Discovery Studio software predictions. The primers were designed using software Primer 3, with the amplification efficiency of the target and reference genes certified using the same program. The relative expression levels of the target genes were normalized internally to the 16S rDNA level and quantified using the 2^−ΔΔCT^ method with β-actin as the housekeeping gene.^23^ For each gene, the reference samples were taken as having an expression level of 1.0, and the data for other samples were expressed as the fold increase of the mRNA level over the reference sample. The sequences of the primer pairs are listed in Table 1.

**Table 1:** Sequence of primers used in the qRT-PCR analysis

Gene name	Primer sequence	Product size
Prkag2	F:5′-GCAGAAAAACTACAGCAGAAGACTC-3′	123 bp
	R:5′-CTTGCAACGTAGTGTCGAAGAC-3′	
Keap1	F: 5′-CTGGTATCTGAAACCCGTCTA-3′	117 bp
	R:5′-TGGCTTCTAATGCCCTGA-3′	
β-Actin	F: 5′-CTGTCCCTGTATGCCTCTG-3′	218 bp
	R:5′-ATGTCACGCACGATTTCC-3'	

### Statistical analysis

All statistical analyses were performed using the SPSS10.0 (SPSS, Inc., Chicago, USA) statistical package. *P* < 0.05 was considered statistically significant. All results are presented as the mean ± standard deviation (S.D.).

## Results

### Optimization of the enzymatic hydrolysis conditions

**Fig. 1:**
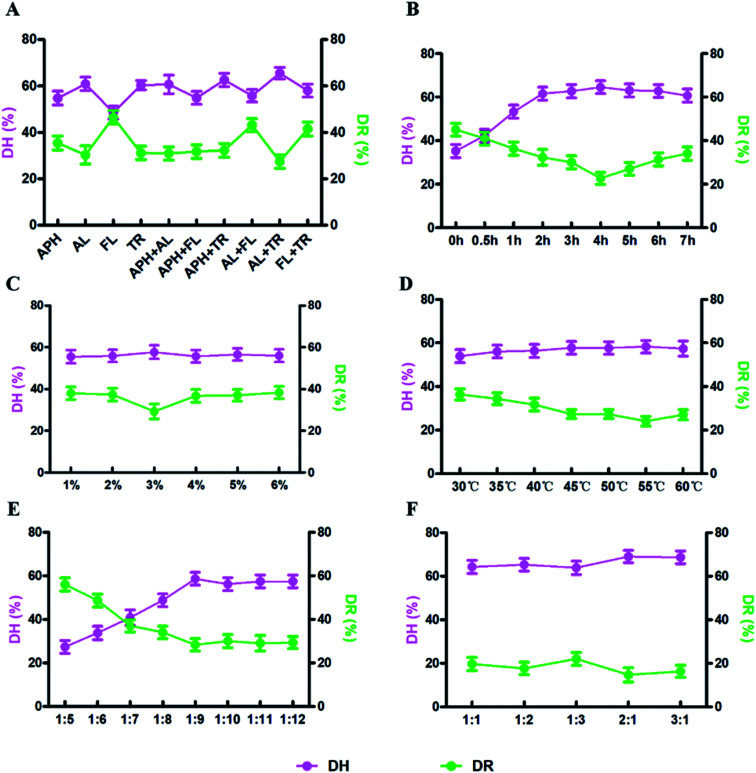
Effects of the different factors on enzymatic treatment. (A) Types of enzyme (APH: animal protein hydrolase, AL: alkaline protease, FL: flavourzyme, TR: trypsin), (B) time, (C) enzyme concentration, (D) temperature, (E) solid-to-liquid ratio, (F) enzyme ratio.

**Fig. 2:**
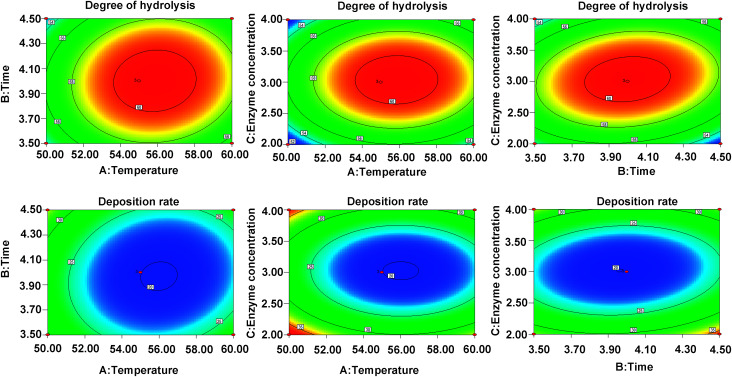
The contour plots demonstrating the effects of temperature (A), enzymolysis time (B) and enzyme concentration (C) on the DH (Y1) and DR (Y2).

DH and DR indexes are used as the indexes to measure the enzymatic hydrolysis efficiency, the combination with the highest DH and the lowest DR is regarded as the most efficient one (Fig. 1). In this study, the optimal enzymolysis condition is confirmed *via* single factor experiment (Fig. 1) and response surface experiment (Fig. 2, ESI Tables S1 and S2†), that is trypsin and alkaline combination at the ratio of 2 : 1, 4 h hydrolysis under 55 °C, the enzyme concentration was 3% and the solid–liquid ratio was 1 : 9. In this condition, the DH and DR reached 60.7% and 19.8%, respectively.

### Polypeptide composition in hydrolysate

**Fig. 3:**
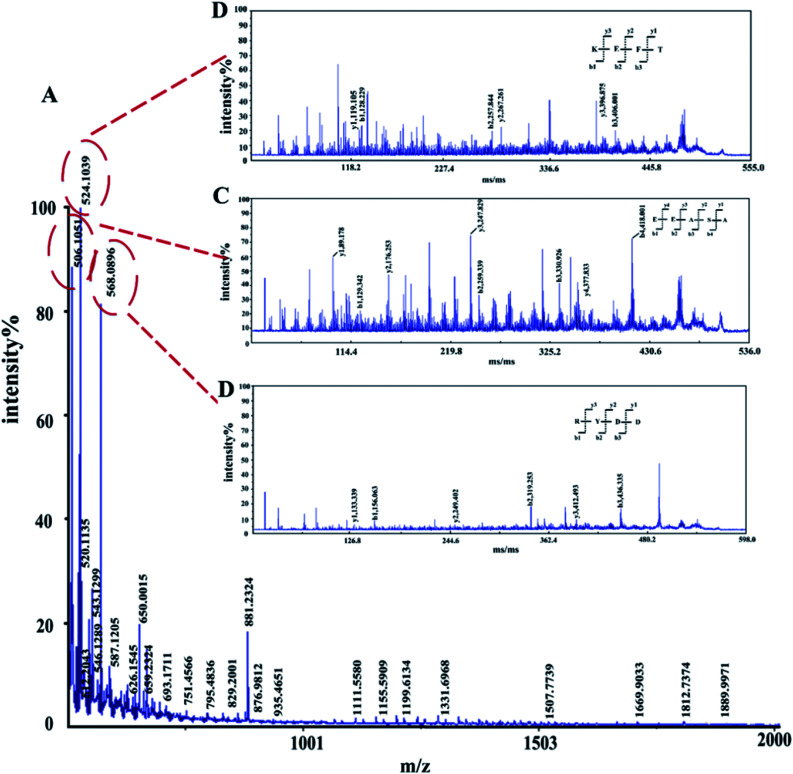
MALDI-TOF/TOF tandem mass spectra of polypeptides. (A) First-order mass spectra of the enzymatic peptides. (B)–(D) Secondary mass spectrometry.

The amino acid sequence of the enzymatic hydrolysate was determined by MALDI TOF/TOF-MS. In the *m*/*z* 0–2000 range, 403 peaks were detected, and three peaks showed higher intensities than the others, corresponding to *m*/*z* 506.1051, *m*/*z* 524.1039 and *m*/*z* 568.0896 (ESI Table S3†), which were predicted to be KEFT (Lys-Glu-Phe-Thr), EEASA (Glu-Glu-Ala-Ser-Ala) and RYDD (Arg-Tyr-Asp-Asp) (Fig. 3).

### Polypeptide function prediction using Discovery Studio software

The potential activity of the three polypeptides was screened *via* the Discovery Studio software reverse search function, and the results are shown in Table 2. KEFT can bind to 5dad (Keap1) (3.56 fit-value), 1uk4 (replicase polyprotein) (3.35 fit-value) and 1m4d (aminoglycoside 2′-*N*-acetyltransferase) (3.25 fit-value); EEASA can bind to 5dad (3.69 fit-value), 1uk4 (3.60 fit-value) and 1o86 (angiotensin-converting enzyme) (3.33 fit-value); and RYDD can bind to 5dad (3.62 fit-value), 1jj7 (antigen peptide transporter1) (3.41 fit-value) and 1uk4 (3.37 fit-value). Keap1 is an important regulator of cellular oxidative stress, and all three domain peptides can bind to Keap1 with the highest fit-value. Therefore, we presumed that Keap1 is a potential target.

**Table 2:** The results of potential targets screening with high fit values

Name	Pharmacophore number	Pharmacophore	Fit value	Biological function
KEFT	5dad-02	Kelch-like ECH associated protein 1(Keap1)	3.566	Oxidation^25^
	1uk4-06	Replicase polyprotein 1ab	3.356	Acute respiratory syndrome^44^
	1m4d-05	Aminoglycoside 2′-*N*-acetyltransferase	3.25	Confer resistance to aminoglycosides^45^
	1o86-02	Angiotensin-converting enzyme	3.22	Treat hypertension^46^
	1tyr-06	Transthyretin	3.17	Tumor and inflammation^43^
EEASA	5dad-01	Kelch-like ECH associated protein 1	3.69	Oxidation^25^
	1uk4-07	Replicase polyprotein 1ab	3.60	Acute respiratory syndrome^44^
	1o86-01	Angiotensin-converting enzyme	3.33	Treat hypertension^46^
	1tyr-08	Transthyretin	3.29	Tumor and inflammation^43^
	3lbj-09	Mdm4 protein	3.21	Cancer^47^
	2dq7-07	Tyrosine-protein kinase Fyn	3.11	Regulation of cell growth and survival adhesion^48^
RYDD	5dad-01	Kelch-like ECH associated protein 1	3.62	Oxidation^25^
	1jj7-09	Antigen peptide transporter 1	3.41	Binding of peptide^49^
	1uk4-06	Replicase polyprotein 1ab	3.37	Acute respiratory syndrome^44^
	1m48-04	Interleukin-2	3.28	Regulation of the immune response^50^
	3beg-01	SRSF protein kinase 1	3.18	Cell growth and tumor^51^
	3d9k-08	Protein SCAF8	3.14	mRNA processing^52^

**Fig. 4:**
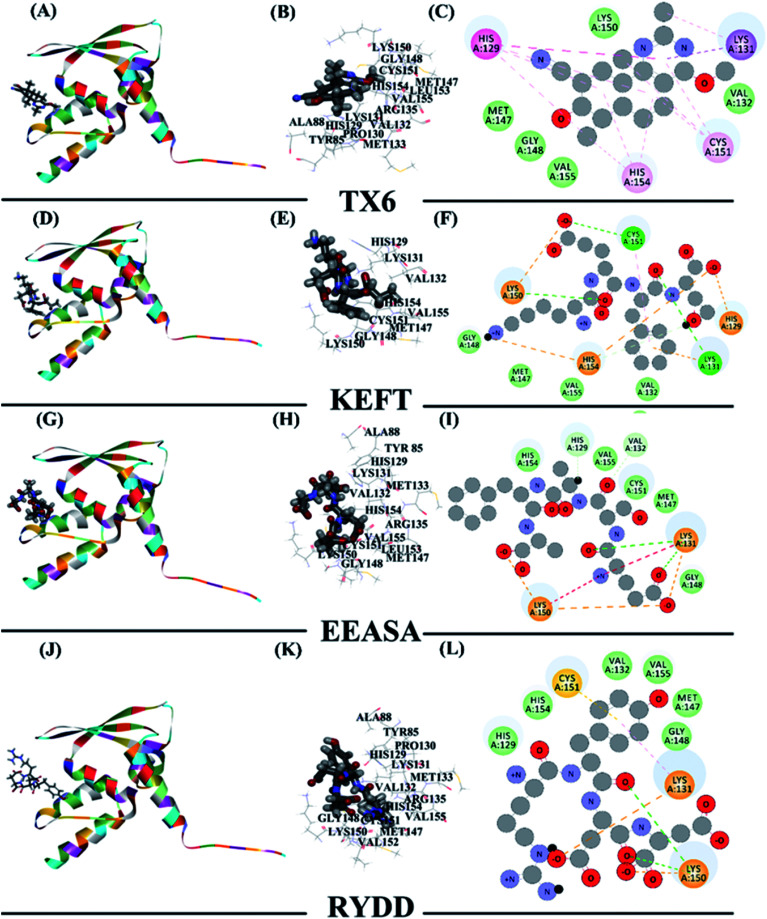
The interaction of polypeptides with Keap1. (A), (D), (G), and (J) the protein and polypeptide are integrally bound. The polypeptide is compatible with the protein active site. (B), (E), (H), and (K) the polypeptide binds at the active site. (C), (F), (I), and (L) amino acid residue 2D diagram.

To explain how the three polypeptides bind to the Keap1 protein, the molecular docking function in the Discovery Studio software was used. The molecular docking results should be based on the receptor active site, receptor-ligand-CDOCKER interaction energy (CIE), number of interacting amino acid residues and other information to determine the extent of binding^24^ (Table 3). Previous studies have reported that TX6 binds to the Keap1 protein to inhibit the activity of Keap1 as well as the Nrf2/ARE signaling pathway.^25^ Therefore, we chose TX6 as a reference to evaluate the degree of peptide and Keap1 protein binding, and the CIE values of KEFT, EEASA and RYDD were 66.6, 76.2 and 71.3, respectively, while the CIE value for the reference TX6 was 26.1. TX6 and the peptides KEFT, EEASA and RYDD have similar docking sites that are located at the same active site (Fig. 4A, D, G and J). The molecular docking study showed that TX6 established 10 π bonds with 5 Keap1 amino acid residues, and the remaining seven amino acid residues interacted with Keap1 by VDW (Fig. 4B and C). There are 14 amino acid residues that interact with KEFT and Keap1, and the number of amino acid residues involved in hydrogen bonding (HB) and VDW is 3 and 6, respectively. KEFT interacts with Met147, Lys131 and Cys151 to form 5 HBs (Fig. 4E and F). As shown in Fig. 4H and I, there are 12 amino acid residues associated with Keap1 and EEASA interactions, and 6 HBs interact with Lys131, Arg135, Met147, His129, Cys151 and Lys150 to participate in 6 VDW amino acid residues. There are 14 amino acid residues bound to RYDD and Keap1, among which 2 are HBs; these are Lys150 and Asp87, respectively. In addition, 4 amino acids are involved in VDW (Fig. 4K and L). All of these results indicate that KEFT, EEASA and RYDD can bind to the Keap1 protein.

**Table 3:** The results of molecular docking

Abbreviation	Name	Pose number	-CIE	Number of HB	Number of π-bond	Number of amino acid residues interacting
TX6	(6aS,7S,10aS)-8-hydroxy-4-methoxy-2,7,10a-trimethyl-5,6,6a,7,10,10a-hexahydrobenzo[h]quinazoline-9-carbonitrile	1	26.094	—	10	12
KEFT	Lys-Glu-Phe-Thr	1	66.6412	5	—	14
EEASA	Glu-Glu-Ala-Ser-Ala	1	76.1739	6	—	12
RYDD	Arg-Tyr-Asp-Asp	1	71.3115	4	—	14

### Effect of antioxidant activity

**Fig. 5:**
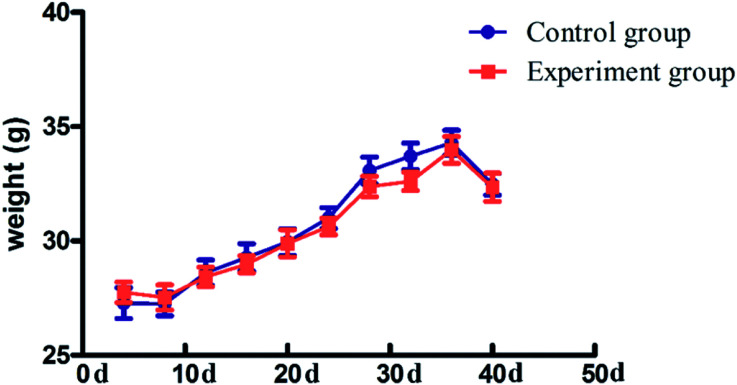
The body weight of mice in the control group and experiment groups. The data are expressed as the mean ± SD, *n* = 12. ANOVA was used to assess the differences. *P < 0.05; **P < 0.01, compared with the control group.

**Fig. 6:**
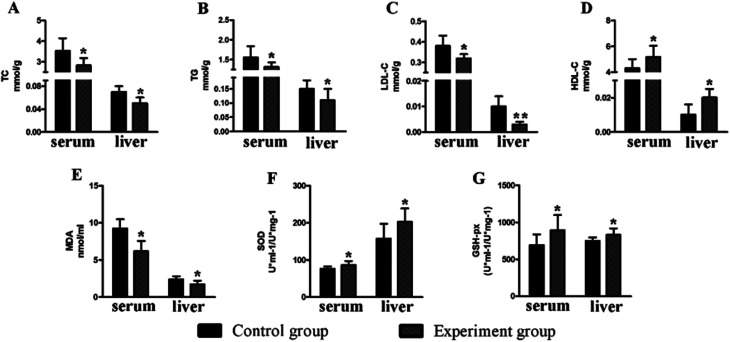
The effects of the polypeptide on the physiological and biochemical indexes of mice. (A) The TC content (B) The TG content (C) the LDL-C content (D) the HDL-C content (E) the MDA content (F) the SOD activities (G) the GSH-Px activities; the data are expressed as the mean ± SD, *n* = 12. ANOVA was used to assess the differences, *P < 0.05; **, *P* < 0.01, compared with the control group.

There was no significant change (*P* > 0.05) in the body weight of mice in the experimental group and control group (Fig. 5). Compared to the control group, the levels of serum TC (2.83 ± 0.35 mmol g^−1^, *P* < 0.05), TG (1.22 ± 0.11 mmol g^−1^, *P* < 0.05) and LDL (0.32 ± 0.02 mmol g^−1^, *P* < 0.05) in the experiment group were significantly reduced, while the HDL level was increased (5.18 ± 0.87 mmol g^−1^, *P* < 0.05). The situation in the liver is similar to that of the serum (Fig. 6), with reduced TC (0.05 ± 0.01 mmol g^−1^, *P* < 0.05), TG (0.11 ± 0.04 mmol g^−1^, *P* < 0.05), and LDL (0.01 ± 0.01 mmol g^−1^, *P* < 0.01) and increased HDL (0.02 ± 0.005 mmol g^−1^, *P* < 0.05).

Compared to the control group, the levels of serum GSH-Px (893.11 ± 206.58 U ml^−1^/U mg^−1^, *P* < 0.05) and SOD (86.29 ± 11.18 U ml^−1^/U mg^−1^, *P* < 0.05) in the experimental group were significantly increased, while the MDA level (6.19 ± 1.38 nmol ml^−1^, *P* < 0.05) was significantly decreased. The situation in the liver is similar to that of the serum (Fig. 6), with increased GSH-Px (831.15 ± 88.92 U ml^−1^/U mg^−1^, *P* < 0.05) and SOD (202.96 ± 35.76 U ml^−1^/U mg^−1^, *P* < 0.05) and decreased MDA levels (1.72 ± 0.51 nmol ml^−1^, *P* < 0.05).

### Characterization of the mRNA expression level

**Fig. 7:**
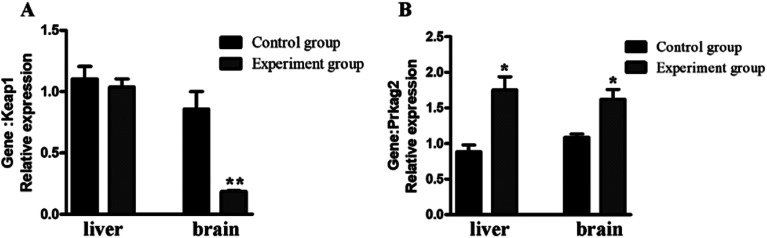
Relative expression of antioxidant genes in the mouse liver and brain. *P < 0.05; **, *P* < 0.01, compared with the control group.

Keap1 is an important regulator of cellular oxidative stress. The γ2 regulatory subunit of AMP-activated protein kinase, which is encoded by the gene *Prkag2*, is a heterotrimeric Ser/Thr kinase that acts against oxidative damage.^26^ To verify the antioxidation effect at the mRNA-level, two anti-oxidative related genes were selected for qRT-PCR analysis (Fig. 7). The results showed that Prkag2 was significantly up-regulated in the liver (*P* < 0.05) and brain (*P* < 0.05), whereas Keap1 was only significantly down-regulated only in the brain (*P* < 0.01) after treatment.

## Discussion

In this study, we firstly optimized the enzymatic hydrolysis conditions *via* traditional response surface experiments, and the results is shown in Fig. 1 and 2. The use of enzymatic hydrolysis is often considered as an appropriate and useful method for maintaining the nutritional value of proteins and improving the functional properties of them.^27^ The process depends on several factors including enzyme type, enzyme concentration, ratio of enzyme, solid-to-liquid ratio, temperature and enzymolysis time.^28^ These factors cooperatively affect the enzyme activity and thus make the hydrolysis process more controllable. Several enzymes, such as alkaline protease, trypsin, papain, pepsin, flavourzyme, animal protein hydrolase, and thermolysin, have been widely used in fish proteins hydrolyzing.^29^ In this experiment, the alkaline protease and trypsin were chosen *via* single factor experiment, and then Box-Behnken design (BBD), a popular tool to design response surface, was used to optimize the hydrolysis conditions.^30–32^

The peptides in the dark muscle hydrolysate were subsequently sequenced after enzymolysis, and the dominant polypeptides were predicted to bind to an antioxidant protein (Keap1) (Fig. 4, Tables 2 and 3). The subsequent animal experiments showed that MDA in the treatment group were significantly reduced and SOD and GSH-Px were significantly increased (Fig. 6) compared to the control group. In addition, Keap1 was down-regulated in the treatment group, while Prkag2 was significantly up-regulated. All of these results indicate that the hydrolysates derived from tuna dark muscle are promising candidates for antioxidation and blood lipid control.

The research findings showed that the serum TC, TG and LDL-C levels were significantly decreased in the experimental group and that the serum HDL-C level was increased, and similar results were obtained in the liver. Oxidative stress has a negative effect on the body, which is induced by free radicals and is considered to be an important factor leading to diabetes mellitus and cardiovascular disease, causing a series of harmful biochemical reactions that lead to dyslipidemia.^33^ Various active substances not only act as an antioxidant but also regulate blood lipid metabolism. The *Panax quinquefolius* polysaccharide peptide showed an anti-oxidant effect and can be used to reduce blood glucose and blood lipid in diabetic mice.^34^ Goose oil improves the antioxidant capacity, ameliorates blood lipid metabolism and resists atherosclerosis in mice.^35^ Research has shown that 13.6% of dongchunghacho rice supplementation to polished rice has antioxidant potential and is hypocholesterolemic as well as hypoglycemic in STZ-induced diabetic rats.^36^ However, the link between oxidative stress and lipid metabolism is not clear. On the one hand, hydrolysate treatment significantly increased the activities of GSH-Px and SOD and decreased the MDA levels as well as returned blood lipid metabolism to normal *via* free radical scavenging. On the other hand, research has shown that the serum TG levels influence the plasma MDA concentrations.^37^ Thus, we hypothesize that TG can improve blood lipid disorders by increasing the body's antioxidant capacity. In addition, reverse docking results show that RYDD and KEFT can bind to the low-density lipoprotein receptor (2.66 and 2.63 fit-value, respectively). The low-density lipoprotein receptor, which can bind LDL, is the major cholesterol-carrying lipoprotein in plasma and transports cholesterol into cells by endocytosis to regulate hyperlipidemia (ESI Table S4†). LDL, known as “bad cholesterol”, plays a role in transporting endogenous cholesterol and cholesteryl ester in plasma and its concentration is associated with an increased prevalence of atherosclerosis. Degradation of LDL occurs *via* the LDL receptor pathway. In this experiment, the binding of polypeptide to KEAP1 is relatively higher than to the low-density lipoprotein receptor, which prevents the binding of polypeptide to the LDL receptor. Therefore, the LDL receptor binds to LDL and LDL is degraded through the LDL receptor pathway, which may explain the lipid-lowering function.

Reverse docking is a powerful tool that is used for drug repositioning and rescue.^10,38^ From our results, we used reverse docking to find an antioxidant-related protein that can affect the oxidative stress system. In response to active oxygen damage, the body has formed a complex oxidative stress response system, the coordination response is regulated by the antioxidant responsive element (ARE) of these protective genes in the upstream regulation region.^39,40^ Nrf2 is a critical factor for regulating the intracellular expression of many antioxidants.^41^ Keapl-Nrf2/ARE, as an effective antioxidant regulation pathway, has played an important role in cellular defense and reduces the oxidative stress of the human body.^42^ Inhibition of Keap1 activity activates antioxidant stress of the Nrf2/ARE signaling pathway. In this study, the value of -CIE of KEFT, EEASA and RYDD were higher than that of TX6, which acted as a reference to evaluate the binding degree between polypeptides and the Keap1 protein, indicating better binding of KEFT, EEASA and LCGEC with Keap1 than TX6. Thus, the polypeptide can inhibit the activity of Keap1and activate the antioxidant stress of the Nrf2/ARE signaling pathway. In addition, Prkag2 was significantly increased in the polypeptide treatment group. Prkag2 is involved in the AMPK pathway, and AMPK is a heterotrimeric Ser/Thr kinase that acts against oxidative damage. Therefore, we believe that the antioxidant activity of hydrolyzate can be achieved by affecting multiple pathways.

From the reverse docking results, polypeptide functions were predicted, such as anti-hypertension, cell growth regulation, anti-tumor activity, and immune system regulation. In this study, KEFT and EEASA bind to 1o86 (angiotensin-converting enzyme) (3.22 and 3.33 fit-value, respectively), which may reduce the formation of angiotensinIIand increase the activity of bradykinin. This function can effectively relieve high blood pressure, heart failure, diabetes, hypertension and other diseases.^17^ KEFT and EEASA also can bind to 1tyr (transthyretin) (3.17 and 3.29 fit-value, respectively), which is an inhibitor of monocyte and endothelial cell interleukin-1 production; therefore, the combination of polypeptides and transthyretin can effectively promote interleukin-1 production and exhibit anti-tumor and immune regulation activity.^43^

In this study, the *in silico* test showed that the three peptides (KEFT, EEASA, RYDD) in the hydrolysate showed antioxidant activity *via* binding to protein Keap1, and the subsequently animal experiments confirmed the prediction. Therefore, it provides a high throughput strategy to screen new function of hydrolysate. However, due to a lack of the animal experiment with the synthesized single peptide (KEFT, EEASA, RYDD), it is hard to indicate whether they actually possess antioxidant activity, as well as which peptide play a key role in *in vivo* antioxidant activity. This shortage of the strategy needs to be considered in the further applications.

## Conflicts of interest

The authors declare no competing financial interest.

## Supplementary Material

RA-008-C8RA00889B-s001
