# Supplementary material for: In silico analysis and in vivo tests of the tuna dark muscle hydrolysate anti-oxidation effect
Source: RSC Adv. 2018 Apr 17;8(25):14109–19. doi: 10.1039/c8ra00889b (PMC9079911; doi:10.1039/c8ra00889b)
Supplement: RA-008-C8RA00889B-s001 [file RA-008-C8RA00889B-s001.pdf]

Electronic Supplementary Material (ESI) for RSC Advances.
This journal is © The Royal Society of Chemistry 2018

In-silico analysis and in-vivo tests of the tuna dark muscle hydrolysate anti-oxidation effect

Jiaojiao Han[1], Shasha Tang[1], Yanyan Li[2], Wei Bao[1], Haitao Wan[1], Chenyang Lu[1], Jun Zhou[1*], Ye Li[1], Lingzhi Cheong[1], Xiurong Su[1*]

[1] School of Marine Science, Ningbo University, Ningbo, China

[2] College of Agriculture and Life Sciences, Cornell University, Ithaca, USA

*** Corresponding author**:

Prof., Xiurong Su

School of Marine Science, Ningbo University, 818 Fenghua Road, Ningbo, China

Tel/Fax: +86 574 87608368. E-mail address: suxiurong_public@163.com

Dr. Jun Zhou

School of Marine Science, Ningbo University, 818 Fenghua Road, Ningbo, China

Tel/Fax: +86 574 87608368. E-mail address: zhoujun1@nbu.edu.cn

Method

Single factor experiment

The degree of hydrolysis (DH) is defined as the percentage of nitrogen in the supernatant:

$$\mathrm{DH}\ (\%) = \frac{N_s}{N_{all}} * 100\%$$

where N_s is the nitrogen content in the supernatant, and N_{all} is the total nitrogen content in the sample with same weight.

The deposition rate (DR) is defined as the percentage of sample dry weight in the precipitate:

$$\mathrm{DR}\ (\%) = \frac{Dry\ Weight_p}{Dry\ Weight_{sample}} * 100\%$$

where $Dry\ Weight_p$ is the dry weight of the precipitate, and $Dry\ Weight_{sample}$ is the dry weight of the sample.

The DH and DR are measured as described below: 10 g dark muscle, a certain amount water and enzyme were added in jacketed 250 mL glass vessels equipped with a stirrer (IKA C-MAG HS 7, IKA, Germany) and connected to a circulating water bath (IKA ICC basic, IKA, Germany) to control the temperature. After enzymolysis, the mixture was heated in a boiling water for 10 min to inactive the enzyme, and subsequently centrifuged at 4000 rpm at 4°C for 10 min after cool to the room temperature. The DH was estimated according to the method of Anwar Noman[1] with some modifications. The nitrogen content was determined as the Kjeldahl method discribed[2]. The residual solid particles after centrifugation and the sample with sample weight (10 g) were heated under 80 °C for 48 h until the dry weight data reached stable.

The DH and DR were used as the indexes for optimal enzymatic hydrolysis screening, including enzyme type, enzyme concentration, enzymolysis time, enzyme ratio, enzymolysis temperature and the solid-to-liquid ratio. The enzyme was selected among animal protein hydrolase (200,000 U/g), alkaline protease (200,000 U/g), flavourzyme (200,000 U/g) and trypsin (4000 U/g) (Guangxi Nanning Pang Bo Biological Engineering Co., Ltd, Nanning, China).

Response surface design

Based on the single factor experiment, the optimal conditions of each factor were used as experimental factors. The DH and DR were used as the response values. The Box-Behnken central combination principle was used to design and optimize the experiment to determine the optimum conditions of enzymatic hydrolysis. In addition, the three factors and three levels were designed to optimize the hydrolysis conditions of dark muscle.

Single factor experiment results

In the single factor experiment, DH and DR indexes are used as the indexes to measure the enzymatic hydrolysis efficiency, the combination with the highest DH and the lowest DR is regarded as the most efficient one. In this study, the optimal enzymolysis condition is: the optimal enzyme formula is trypsin and alkaline at the ratio of 2:1, the hydrolysis time was 4 h, the enzyme concentration was 3%, the heat temperature was 55 °C and the solid-liquid ratio was1:9 (Figure 1).

Optimization by response surface design

According to the single factor experiment, three individual parameters and three levels in the Box-Behnken design, the hydrolysis degree was chosen as the response value, and the hydrolysis temperature (A), hydrolysis time (B) and content of enzyme (C) were selected to respond to the surface experiment (Supplementary Table S1). ANOVA of the data was performed using Design Expert 8.0.5 software. The second-order polynomial equation (Eq (3) and (4)) obtained by fitting the three factors was as follows:

$$Y1 = 60.56 + 1.125 * A - 0.045 * B + 0.31 * C + 0.3775 * A * B + 0.1425 * A * C + 0.9575 * B * C - 3.32125 * A\text{^}2 - 2.70125 * B\text{^}2 - 4.39125 * C\text{^}2 \quad (3)$$

$$Y2 = 20.02667 - 2.2525 * A + 0.67625 * B - 0.53375 * C - 0.9375 * A * B + 0.0975 * A * C - 1.115 * B * C + 5.544167 * A\text{^}2 + 4.101667 * B\text{^}2 + 9.791667 * C\text{^}2 \quad (4)$$

where A, B, and C are all coded values.

The results of analysis of variance (ANOVA) for each response (P<0.05) and lack of fit analysis (P>0.05) are shown (Supplementary Table S2). The quadratic term coefficients (A^2, B^2, C^2) were all significant on Y1 and Y2, and both had small P-values (P<0.05). The other terms' coefficients were not significant (P>0.05). Thus, the model can be used to predict the DH and the DR under different enzymolysis conditions. In the present study, three-dimensional (3D) response surface plots were generated using Design-Expert 8.0.5 as presented in Figure 2. The slope of the response surface 3D

image reflects the sensitivity of the response value to the factor.

Supplementary Table S1. Results of the response surface experiment

Serial number	Code Value			Actual Value			Y1 (DH%)	Y2 (DR%)
	A	B	C	t(℃)	T(h)	E(%)		
1	-1	-1	0	50	3.5	3	53.29	30.58
2	1	-1	0	60	3.5	3	55.13	28.36
3	-1	1	0	50	4.5	3	53.19	32.86
4	1	1	0	60	4.5	3	56.54	26.89
5	-1	0	-1	50	4	2	51.66	38.59
6	1	0	-1	60	4	2	53.28	33.48
7	-1	0	1	50	4	4	52.13	37.05
8	1	0	1	60	4	4	54.32	32.33
9	0	-1	-1	55	3.5	2	54.6	32.05
10	0	1	-1	55	4.5	2	51.85	36.58
11	0	-1	1	55	3.5	4	53.17	33.49
12	0	1	1	55	4.5	4	54.25	33.56
13	0	0	0	55	4	3	60.12	20
14	0	0	0	55	4	3	60.81	20.01
15	0	0	0	55	4	3	60.75	21.03

Supplementary TableS2. Analysis of variance (ANOVA) for the second-order polynomial mode. *, *P*<0.05; **, *P*<0.01.

Source	Mean squares	F value	*P* value	Source	Mean squares	F value	*P* value
Y1	15.19	45.31	0.0003**	Y2	58.67	128.19	< 0.0001**
A	10.12	30.19	0.0027**	A	40.59	88.68	0.0002**
B	0.02	0.05	0.8347	B	3.66	7.99	0.0368*
C	0.77	2.29	0.1904	C	2.28	4.98	0.0760
AB	0.57	1.70	0.2491	AB	3.52	7.68	0.0393*
AC	0.08	0.24	0.6435	AC	0.04	0.08	0.7847
BC	3.67	10.94	0.0213*	BC	4.97	10.86	0.0216*
A^2	40.73	121.45	0.0001**	A^2	113.49	247.95	< 0.0001**
B^2	26.94	80.34	0.0003**	B^2	62.12	135.71	< 0.0001**
C^2	71.20	212.31	< 0.0001**	C^2	354.01	773.42	< 0.0001**
Residual	0.34				0.46		
Lack of Fit	0.46	3.16	0.2497		0.76	531.56	0.0019
Pure Error	0.15				0.0014		
R-Squared	0.9879				1.00		
C.V. %	1.05				2.23		

Supplementary Table S3. Peptides measured by MALID-TOF/TOF

Polypeptide sequence	Polypeptide Expected Mr	Polypeptide Caculated Mr	Polypeptide score	Relative Intensity%	Protein Description
KEFT	523.0918	523.2642	22	4.24	Hemoglobin subunit alpha
EEASA	505.0969	505.202	28	3.93	GnRH3
RYDD	567.081	567.2289	17	3.43	somatolactin
VEKE	503.1008	503.2591	31	1.62	gonadotropin hormone LH beta subunit
TIRM	519.1044	519.2839	28	1.47	GnRH3
FPRM	549.0809	549.2733	19	1.34	cytochrome oxidase subunit 1, partial (mitochondrion)
PVALSCHC	828.1918	828.3622	31	0.11	gonadotropin hormone LH beta subunit
MIGGFGNW	880.2256	880.3902	19	0.74	cytochrome oxidase subunit 1, partial (mitochondrion)
YRDFYYKT	1154.5598	1154.5396	31	0.07	gonadotropin hormone LH beta subunit
PPCQLINQTVS	1198.5956	1198.6016	31	0.08	gonadotropin hormone LH beta subunit
TPAAAFQLPPCQ	1242.6197	1242.6067	31	0.08	gonadotropin hormone LH beta subunit
QQGIWAVSLWP	1283.4864	1283.6663	17	0.05	somatolactin
PTVTYPVALSCH	1286.6535	1286.6329	31	0.08	gonadotropin hormone LH beta subunit
VINDDSSHFNR	1302.6432	1302.5953	28	0.06	GnRH3
QHVCTYRDFY	1330.6774	1330.5765	31	0.07	gonadotropin hormone LH beta subunit
PVALSCHCGRCAM	1346.6775	1346.5716	31	0.06	gonadotropin hormone LH beta subunit
TTICSGHCITKDP	1374.6997	1374.6272	31	0.06	gonadotropin hormone LH beta subunit
ITKALPIPSSKSEI	1482.7216	1482.8657	17	0.04	somatolactin

Supplementary Table S4. Potential targets with relatively low fit value screened by DS 2016.

Name	Pharmacophore Number	Pharmacophore	Fit Value	Biological function
RYDD	1AJJ-05	Low-density lipoprotein receptor	2.65912	regulation hyperlipidemia
	1m4d-09	Aminoglycoside 2'-N-acetyltransferase	2.57746	aminoglycoside 2'-N-acetyltransferase activity
	2aio-01	Metallo-beta-lactamase L1 type 3	2.50539	beta-lactamase activity
	3d9m-01	Protein SCAF8	2.31593	RNA binding
	2orj-04	Pulmonary surfactant-associated protein D	2.26166	carbohydrate binding
KEFT	1AJJ-04	Low-density lipoprotein receptor	2.6315	regulation hyperlipidemia
	1jj7-06	Antigen peptide transporter 1	2.62846	ADP binding
	2dq7-1	Tyrosine-protein kinase Fyn	2.56675	ATP binding
	2orj-04	Pulmonary surfactant-associated protein D	2.45056	carbohydrate binding
	3cyq-01	Motility protein B	1.99232	archaeal or bacterial-type flagellum-dependent cell motility
EEASA	1amw-03	ATP-dependent molecular chaperone HSP82	2.5989	ATPase activity, coupled
	2gz7-08	Orf1a polyprotein	2.52791	cysteine-type endopeptidase activity
	3cyq-01	Motility protein B	2.50415	archaeal or bacterial-type flagellum-dependent cell motility
	2orj-04	Pulmonary surfactant-associated protein D	2.25684	carbohydrate binding
	1jj7-09	Antigen peptide transporter 1	2.23903	ADP binding

Reference

1. A. Noman, Y. Xu, W. Q. Al-Bukhaiti, S. M. Abed, A. H. Ali, A. H. Ramadhan and W. Xia, Influence of enzymatic hydrolysis conditions on the degree of hydrolysis and functional properties of protein hydrolysate obtained from Chinese sturgeon (Acipenser sinensis) by using papain enzyme, *Process Biochemistry*, 2018.
2. Kjeldahl Method, *Encyclopedia of Genetics Genomics Proteomics & Informatics*, 2008, 1063-1063.
